# Supplementary material for: Highly reproducible and CMOS-compatible VO2-based oscillators for brain-inspired computing
Source: Sci Rep. 2024 May 21;14:11600. doi: 10.1038/s41598-024-61294-x (PMC11109144; doi:10.1038/s41598-024-61294-x)
Supplement: Supplementary file 1 — Supplementary Information. [file 41598_2024_61294_MOESM1_ESM.docx]

Highly Reproducible and CMOS-compatible VO_2_-based Oscillators for Brain-inspired Computing

Olivier Maher*, Roy Bernini, Nele Harnack, Bernd Gotsmann, Marilyne Sousa, Valeria Bragaglia, and Siegfried Karg*

IBM Research Zurich, Säumerstrasse 4, 8803 Rüschlikon, Zürich, Switzerland

SUPPORTING INFORMATION

**XRR reflectivity measurements**

The XRR curves of Figure S1 are acquired with a Bruker D8 discover diffractometer equipped with a rotating anode generator and analyzed by fitting a simulated curve, based on a multilayer model, to the measured data^1^. XRR profile of Sample K with nominal 50 nm of amorphous VO_2_ deposited on ultrathin 2 nm Si substrate and post annealed to obtain a polycrystalline VO_2_ layer. The XRR analysis demonstrate that the sample has sharp interfaces and no spurious layer between VO_2,_ and Si layers is formed as reported in Table ­S1. Sample L, instead, has nominal 50 nm of amorphous VO_2_ films grown on 50 nm SiO_2_ layer on Si substrate. The XRR analysis reveals that a spurious interfacial layer of ~9 nm with low density of ~3.5g cm^-3^ is detected between the top VO_2_ layer and the SiO_2_. Sample M is similar to Sample K but with 10 nm HfO_2_ interlayer. Also in this case, no spurious interfacial layers are obtained between the top VO_2_ and underneath HfO_2_ layer. All results are summarized in Table S1.


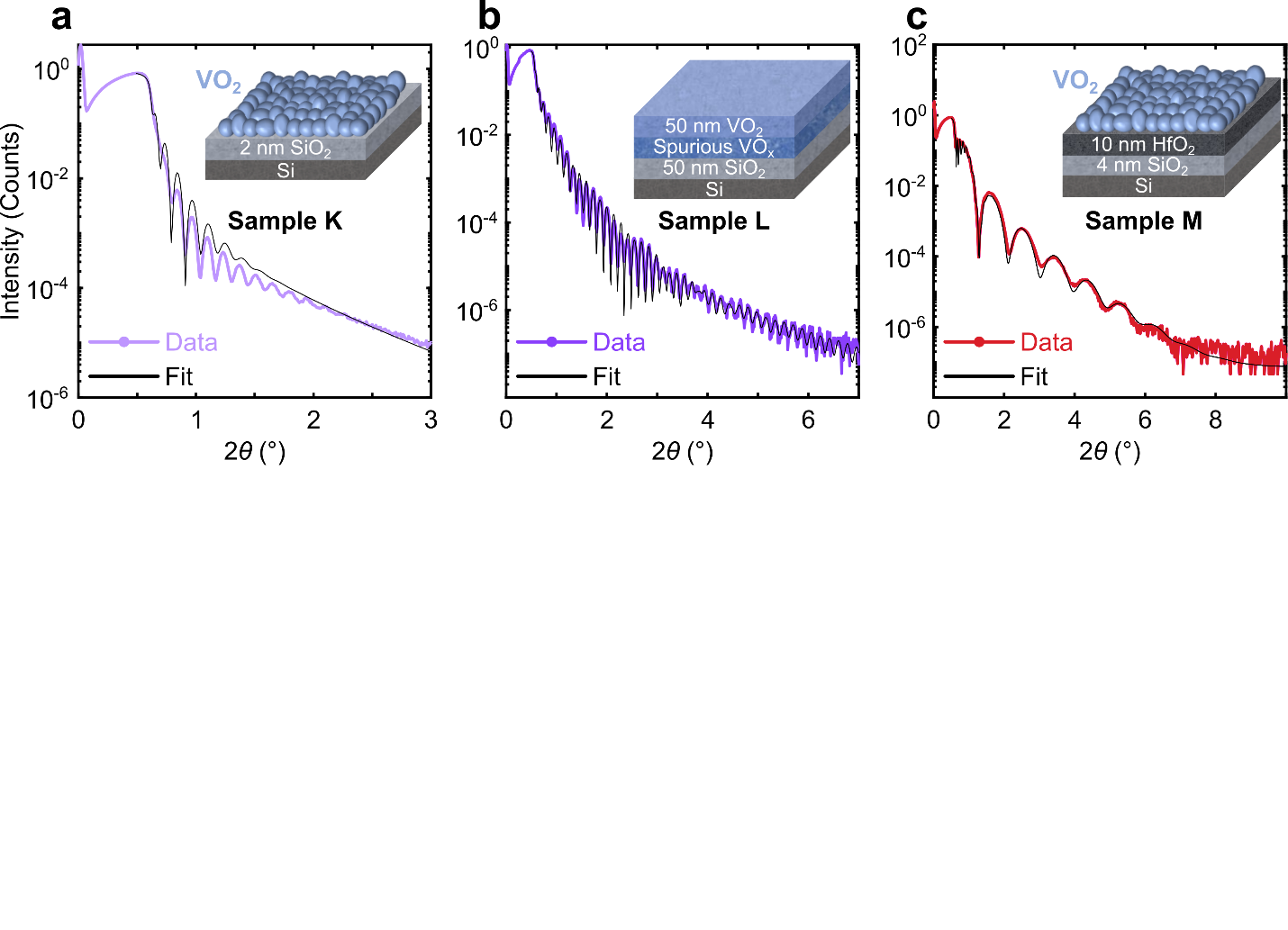


**Figure S1.** **XRR patterns of vanadium oxide samples.** The various stacks are characterized by (**a**) a vanadium oxide layer deposited on 2 nm SiO_2_ after annealing (Sample K); (**b**) vanadium oxide deposited on 50 nm SiO_2_ before annealing (Sample L) and (**c**) vanadium oxide deposited on HfO_2_ layer after annealing (Stack M). Fits are in black while the experimental data are in colored line according to the legend of each figure.

**Table S1. XRR fit analysis for stacks K, L, and M.**

| **Sample** | **Sample Stack** | **Thickness (nm)** | **Roughness (nm)** | **Density (g cm^-3^)** |
| --- | --- | --- | --- | --- |
| **Sample K** | Annealed VO_2_ | 52.6 | 2.9 | 4.6 |
|  | SiO_2_ | 2.0 | 0.5 | 2.3 |
| **Sample L** | Unannealed VO_x_ | 52.8 | 0.9 | 3.8 |
|  | Spurious VO_x_ layer | 9.0 | 0.5 | 3.5 |
|  | SiO_2_ | 48.9 | 0.2 | 2.2 |
| **Sample M** | Annealed VO_2_ | 62.4 | 2.4 | 4.3 |
|  | HfO_2_ interlayer | 9.1 | 0.7 | 11.5 |
|  | SiO_2_ | 3.9 | 0.4 | 2.3 |

**Flash annealing edge effect**

Figure S2 shows the gradual response of the film to the flash annealer across areas going from the center (α) to the edge (δ) of a sample annealed with a flash power of 90 J cm^-2^, an oxygen partial pressure of 20 Pa, and pre-heated at 245 °C. The non-uniform heat distribution generated by the tool lamp introduced edge effects that translated into visible concentric rings on the surface, whose boundaries defined areas where grain size, surface roughness, and vanadium oxidation states changed drastically (see Figure S2c). Close to the center (α), small dense VO_2_ grains were detected, progressively making way to larger V_2_O_5_ microsized grains towards the edges (δ), measured by Raman spectroscopy. In Figure S2a, the grain size and oxidation state variations across sample areas (α, β, and δ) directly relate to the varying R-T response, affecting transition temperatures, resistivities, and hysteresis widths.


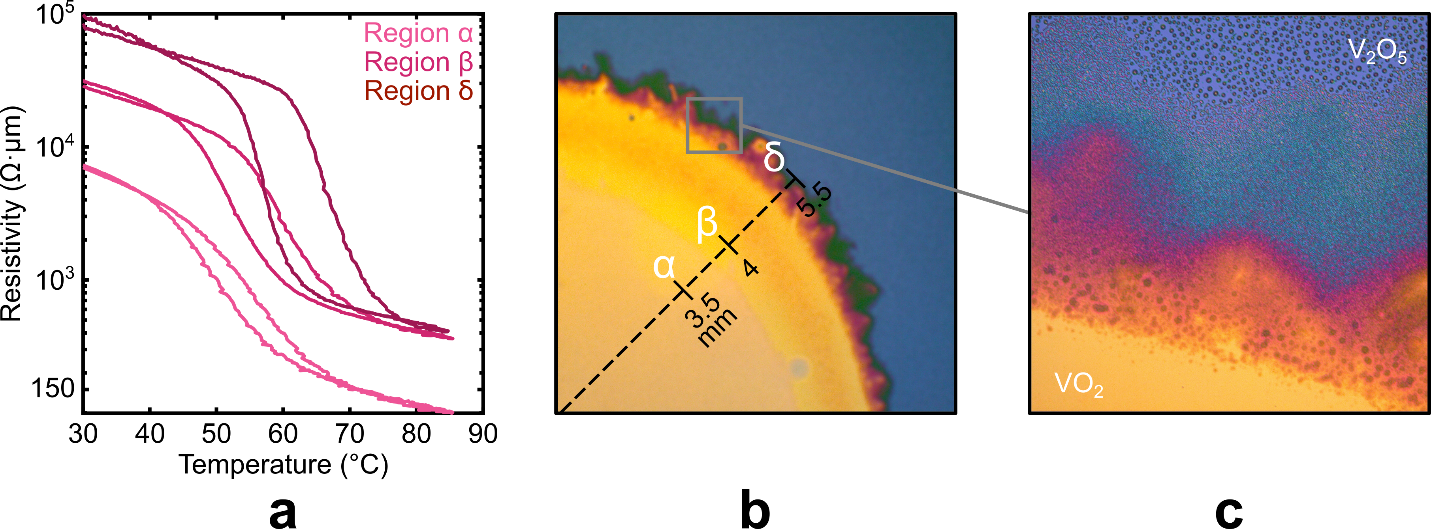


**Figure S2.** **Flash annealing edge effect.** (**a**) 4-probe R-T characteristics measured in different regions (α, β, and δ) of a sample FLA-annealed with the conditions: Pre-flash temp: 245 °C, O_2_ pressure: 20 Pa, Annealing time: 20 ms, Flash power: 90 J cm^-2^. (**b**) Microscopic view of the film showing the varying response to the flash. The progressively larger vanadium-oxide grains are visible in (**c**).

**VO_2_ Growth On Silicon Dioxide (SiO_2_)**

Table S2 summarizes the study results about the SiO_2_ substrate influence on the VO_2_ quality upon annealing with the STA method described in the manuscript. The grain size and surface roughness of three films (samples G-H-J) grown on PECVD, ALD, and thermal SiO_2_ were measured by AFM (see Figure S3), and the quantitative values were extracted with the software Gwyddion.

**Table S2.** **VO_2_ grains statistics of the samples measured in Figure S3.** Data analysis performed with Gwyddion (software version 2.59).

| **STA treatment** | **Sample G** | **Sample H** | **Sample J** |
| --- | --- | --- | --- |
| SiO_2_ growth method | Thermal | PECVD | ALD |
| Average grain diameter size | 37 nm | 41 nm | 39 nm |
| RMS | 2.083 nm | 1.724 nm | 1.909 nm |


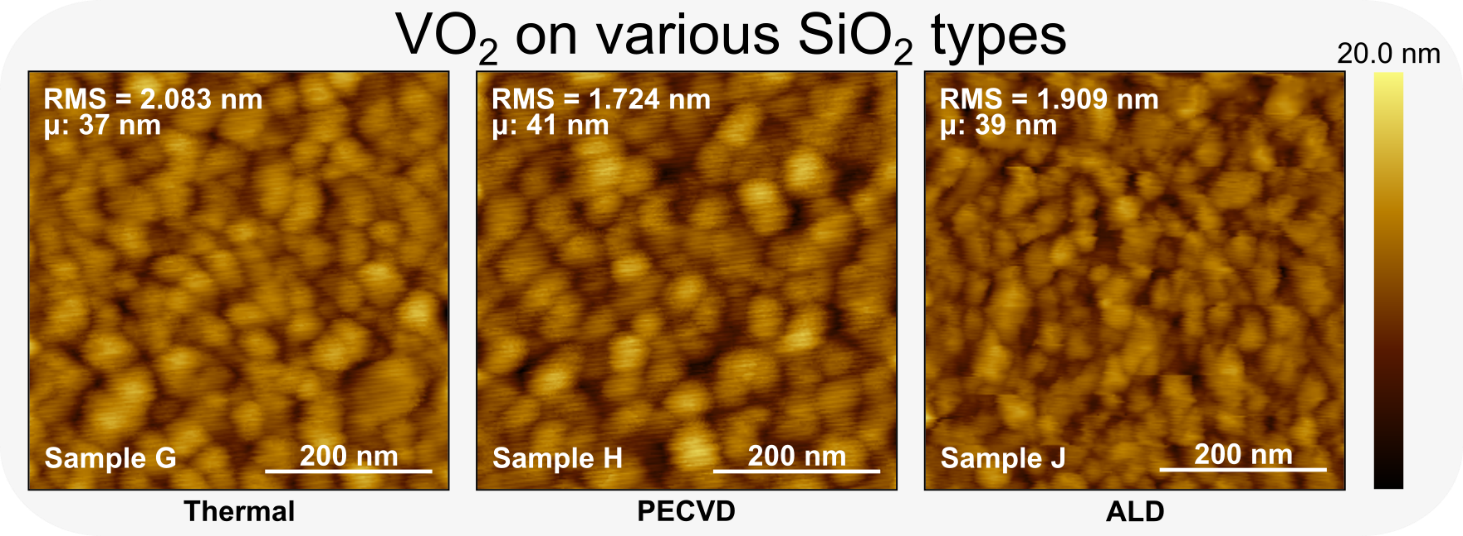


**Figure S3.** **AFM measurements of an STA treated VO_2_ film on a SiO_2_ substrate layer.** The substrate is grown (**a**) thermally (1 μm) (**b**) by PECVD (50 nm), or (**c**) ALD (50 nm). STA: Temp: 520 °C, O_2_ pressure: 5 Pa, Annealing time: 5min.

On all three substrates, the obtained average grain size is around 40 nm, with a nominal variability of less than 4% between samples G, H, and J. The mean surface roughness values are also similar, showing a variation of about 20%.

In Figure S4, a 4-probe measurement of the resistivity against temperature of the VO_2_ films grown on the PECVD and ALD SiO_2_ layers reveals comparable hysteretic behaviors, with 1.5 orders of magnitude drop and a switching temperature close to 68 °C. These findings suggest that the method to grow SiO_2_, with their corresponding differences in topography, purity and dangling bonds, has no or very little impact on the VO_2_ structural properties and transition temperature. However, what stands out in Figure S4 is the remaining variability in the films resistivities measured before and after the phase-transition, similar to the behavior measured for the devices in Figure 6. Measuring the R-T characteristics of VO_2_ grown on all three types of SiO_2_ (ALD, PECVD, and Thermal) leads to nonoverlapping hysteresis curves, i.e. starting and ending at different resistivity values, as in Figures 1 and 2. Variability between VO_2_ devices grown on SiO_2_, regardless of the SiO_2_ deposition technique, is inevitable. Hence, we investigated the growth of VO_2_ grains on different substrate stack by adding an interlayer between the VO_2_ and the SiO_2_.


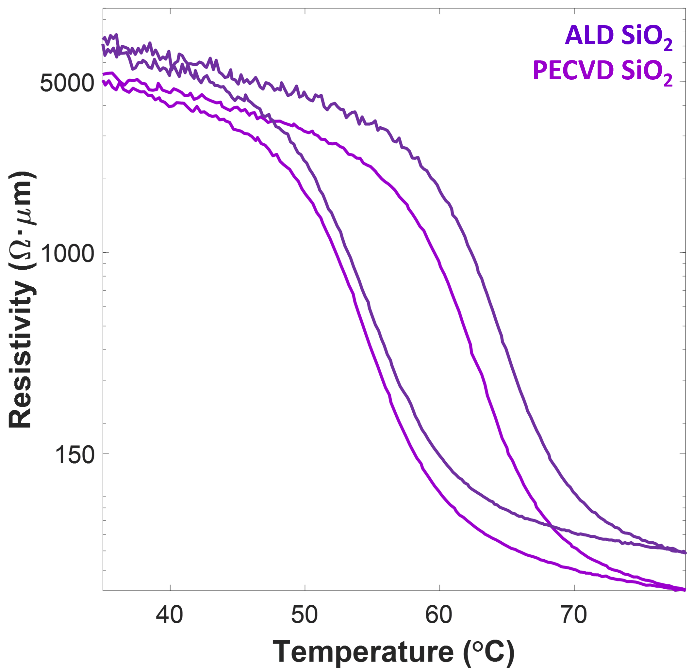


**Figure S4.** **R-T measurement of VO_2_ samples grown on different SiO_2_ types.** The VO_2_ grains are annealed on 50 nm of PECVD or ALD deposited SiO_2_ STA treated: Temp: 520 °C, O_2_ pressure: 5 Pa, Annealing time: 5 min.

**Oscillation failure caused by high variability**

Figure S5a illustrates two VO_2_-based oscillators operating independently without any coupling. The devices exhibit significant variability, evident in both the divergence in their fundamental oscillating frequencies (Figure S5b) and the notable difference in their insulator-to-metallic voltages, varying by more than 12%. When the oscillators are connected like in Figure 7d through a 330 pF capacitor, the expected outcome of stabilizing in an out-of-phase configuration does not occur; the oscillators simply ignore each other and do not lock in frequency (Figure S5d). The significant variation among the devices even makes 'strong coupling' unachievable (demonstrated with 1nF coupling in Figure S5d) since the oscillators continue to resist synchronization (Figure S5e).


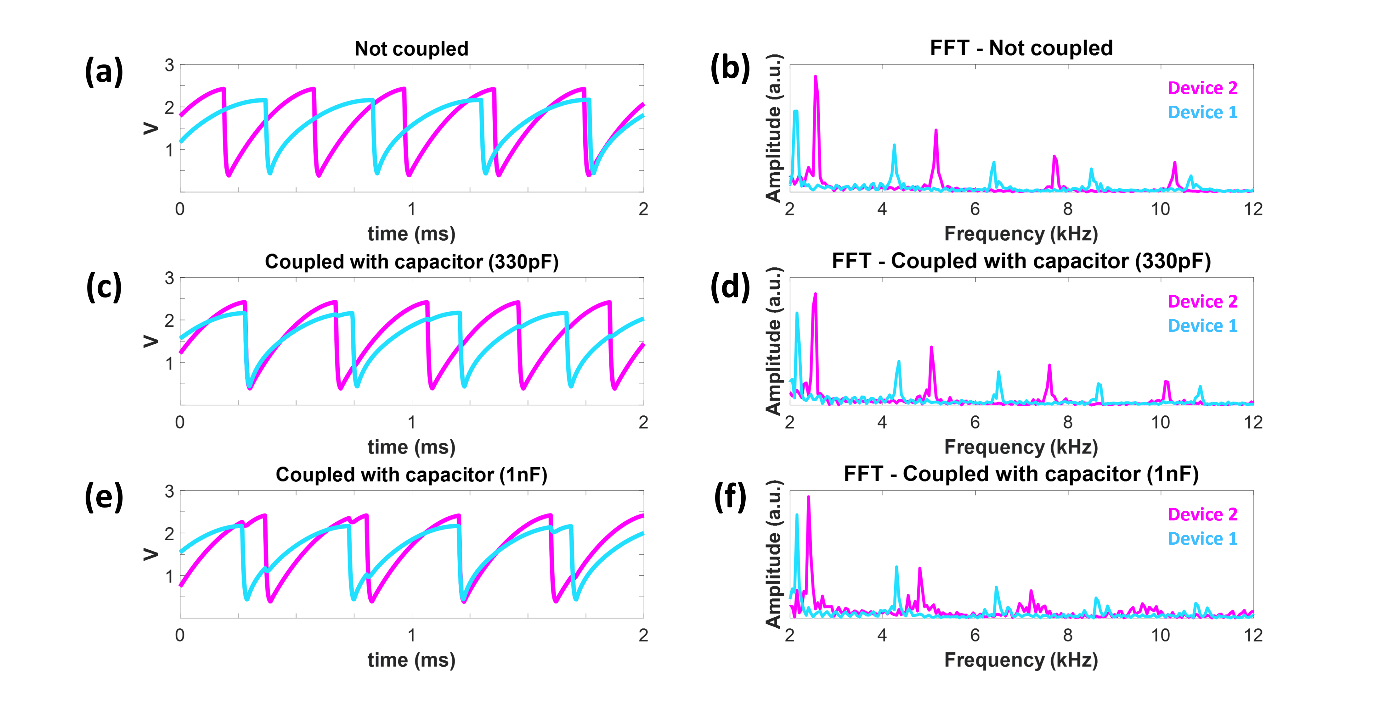
**Figure S5.** **Waveforms and Fast Fourier Transforms (FFT) of two VO_2_-based oscillators coupled and uncoupled.** They show high variability when they are not coupled (**a** and **b**), capacitively (330 pF) coupled (**c** and **d**) and strongly capacitively (1nF) coupled (**e** and **f**). The three coupling schemes show how synchronization is impossible through a capacitor if the oscillators are too different to start with. V_DD_ = 5.5 V. R_s_ = 40 kΩ. C_ext_ = 10 nF. Active area: 100 nm × 100 nm × 60 nm.

When connecting two oscillators with hitgh variability through a dissipative power connection, such as a resistive element, undesirable behaviors arise due to the exchange of current between their output nodes^2^. Figure S6a shows the waveforms of the oscillators when 'weakly coupled' through a 100 kΩ resistor. In this configuration, the network should stabilize in the in-phase configuration, contrary to what is shown in Figure S6a, although synchronization is achieved (Figure S6b). As the coupling resistance is reduced (Figure S6c), thereby increasing the coupling strength, current flows from one oscillator's output node to the other, sometimes preventing it from accumulating enough charge to trigger the phase-transition, as observed in Figure S6c. When the resistance is further decreased (Figure S6e), the phenomenon of 'oscillation death' occurs, meaning the exchange of current becomes too significant. This causes one oscillator to get trapped in the insulator state and the other in the metallic state. When the variability among devices is substantial, finding a balance to address all of these issues simultaneously becomes impossible.


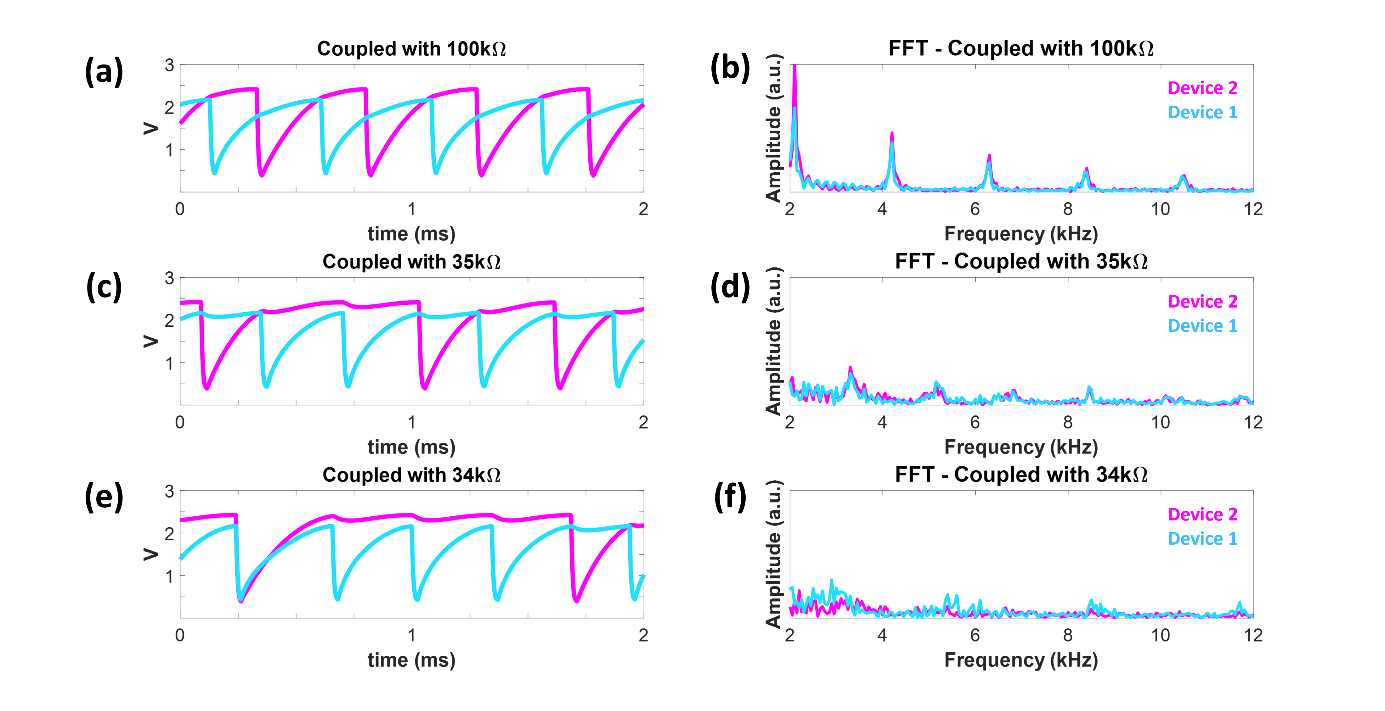
 **Figure S6.** **Waveforms and Fast Fourier Transforms (FFT) of two VO_2_-based oscillators showing high variability when they are weekly coupled.** The coupling is realized through a 100 kΩ resistor (**a** and **b**), and strongly coupled (35 kΩ and 34 kΩ, respectively) coupled (**c** and **d**, and **e** and **f**, respectively). (**a**) shows the unexpected out-of-phase synchronization in the case of weak coupling, while (**c**) and (**e**) show the progressive apparition of the ‘oscillation death’ phenomenon, which is impossible to balance with oscillators exhibiting high variability. V_DD_ = 5.5 V. R_s_ = 40 kΩ. C_ext_ = 10 nF. Cross-section area: 100 nm × 100 nm × 60 nm.

**VO_2_ Grown by RTA recipes and outcomes**

**Table S3.** **RTA tests.** Rapid Thermal Annealing conditions tested to produce smooth granular VO_2_ film on a Si/SiO_2_ substrate. The best reproducible results are observed for the conditions: Temp: 470 °C, O_2_ pressure: 5 Pa, Annealing time: 10 min.

| **RTA Conditions** | | | **Film uniformity** | **Reproducibility** | **Presence of VO_2_** |
| --- | --- | --- | --- | --- | --- |
| Final annealing temperature | O_2_ pressure | Annealing time |  |  |  |
| 430 °C | 5 Pa | 30 sec | Uniform | NO | NO |
| 520 °C | 5 Pa | 30 sec | Uniform | YES | NO |
| 470 °C | 25 Pa | 30 sec | Uniform | YES | NO |
| 470 °C | 25 Pa | 600 sec | Not uniform | NO | NO |
| 470 °C | 5 Pa | 450 sec | Uniform | NO | YES |
| **470 °C** | **5 Pa** | **600 sec** | **Uniform** | **YES** | **YES** |

**
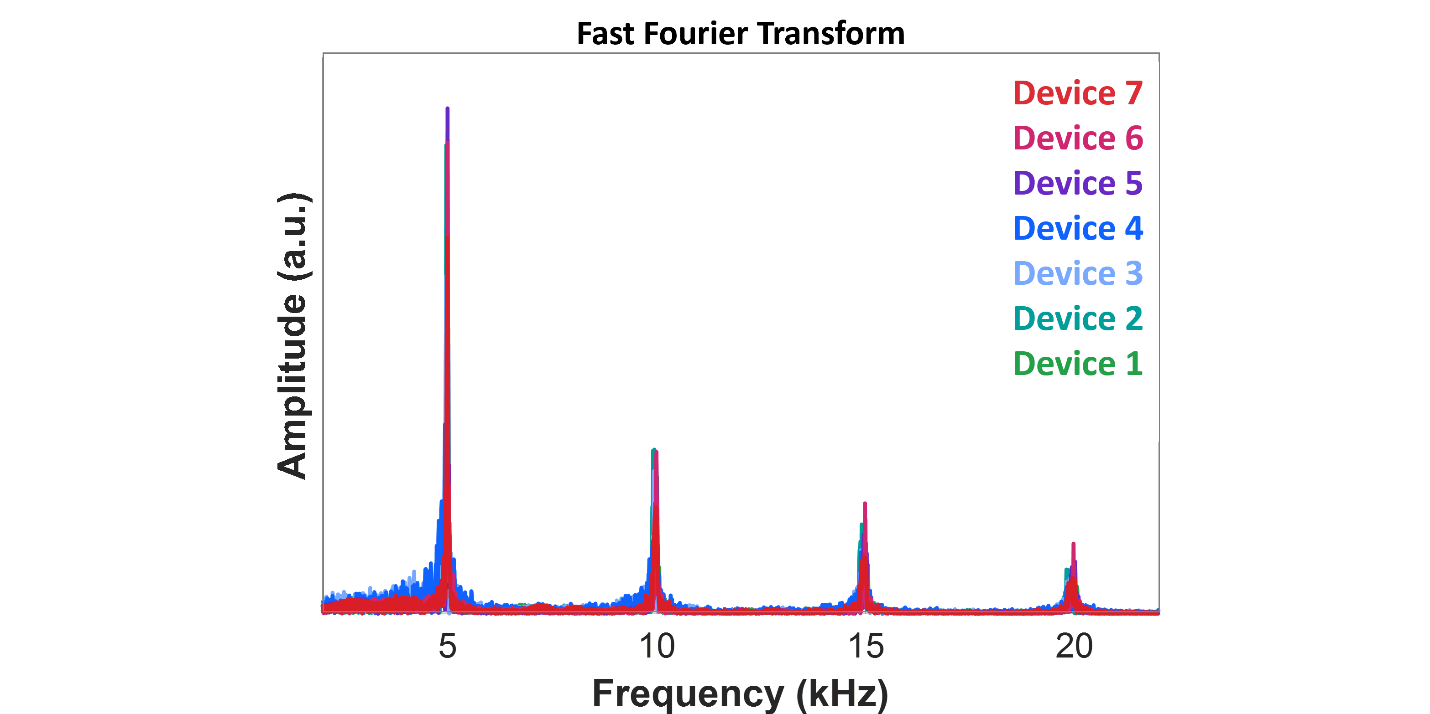
**

**Figure S7.** **Fast Fourier Transforms of the oscillatory response of 7 VO_2_-based crossbar devices.** V_DD_ = 5 V. R_s_ ≈ 40 kΩ. C_ext_ = 10 nF.

**Accuracy of our XRR fitting**

**
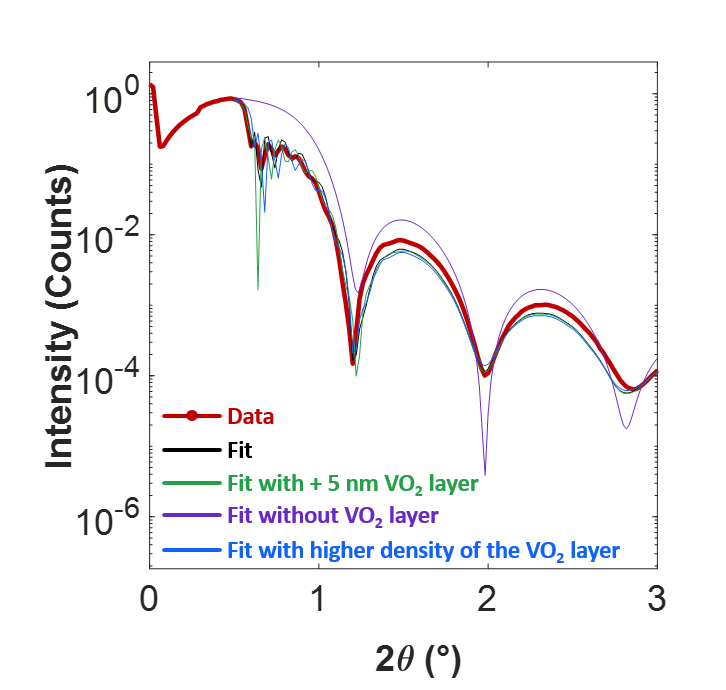
**

**Figure S8.** **Additional XRR fits**. The fits use multilayer models in which the VO_2_ layer is removed (purple curve); the thickness of the VO_2_ layer is increased of 5nm (green curve); the density of the VO_2_ layer is increased to 4.4 g cm^-3^ (blue curve). The original multilayer stack presented in the main manuscript gives the best agreement with the experimental data.

**References**

1. Birkholz, M. *Thin Film Analysis by X-Ray Scattering*. (WILEY-VCH Verlag GmbH & Co.KGaA, Weinheim, 2006).

2. Parihar, A., Shukla, N., Datta, S. & Raychowdhury, A. Exploiting Synchronization Properties of Correlated Electron Devices in a Non-Boolean Computing Fabric for Template Matching. *IEEE J Emerg Sel Top Circuits Syst* **4**, 450–459 (2014).
